# Characteristics and Trends of Pediatric Traumatic Brain Injuries Treated at a Large Pediatric Medical Center in China, 2002–2011

**DOI:** 10.1371/journal.pone.0051634

**Published:** 2012-12-12

**Authors:** Jianbo Shao, Huiping Zhu, Hongyan Yao, Lorann Stallones, Keith Yeates, Krista Wheeler, Huiyun Xiang

**Affiliations:** 1 Department of CT/MRI, Wuhan Children’s Hospital, Wuhan, Hubei, People’s Republic of China; 2 Department of Epidemiology and Health Statistics, School of Public Health and Family Medicine, Capital Medical University, Beijing, People’s Republic of China; 3 Office of Epidemiology, Chinese Center for Disease Control and Prevention, Beijing, People’s Republic of China; 4 Colorado Injury Control Research Center, Department of Psychology, Colorado State University, Fort Collins, Colorado, United States of America; 5 Center for Biobehavioral Health. The Research Institute at National Children’s Hospital, The Ohio State University, Columbus, Ohio, United States of America; 6 Center for Injury Research and Policy, The Research Institute at National Children’s Hospital, The Ohio State University, Columbus, Ohio, United States of America; University of Pittsburgh, United States of America

## Abstract

**Background:**

Pediatric traumatic brain injuries (TBIs) have not been well studied in China. This study investigated characteristics and trends of hospitalized TBIs sustained by Chinese children.

**Methods and Findings:**

We analyzed 2002–2011 hospitalized TBI patients (0–17 years of age) treated at a large pediatric medical center in China. TBIs were defined using the International Classification of Diseases, Tenth Revision (ICD-10) codes. We examined age patterns across external causes of TBIs. We reported the trend of traffic-related TBIs for each year from 2002 to 2011. Of 4,230 TBI patients, 67.1% (95% CI: 65.4%–68.8%) were city residents and 28.8% (95% CI: 26.3%–31.3%) came from rural villages. Males had disproportionately more TBIs than females (65.2% vs. 34.8%). Falls, struck by/against objects, and traffic collisions were the top three external causes of TBIs for all age groups. Falls were the leading cause of TBI for all ages but peaked at 2 years of age. There were 125 TBIs in 0–2 year olds (5.9% of all TBIs in this age group) that were caused by suspected child abuse. Suspected child abuse was significantly more likely to occur in 0–1 year olds. The proportion of traffic -related TBIs increased significantly from 12.99% in 2002 to 19.68% in 2008 but dropped each subsequent year until it reached a level of 8.91% in 2011.

**Conclusions:**

Our study confirms that falls, struck by/against objects and traffic collisions are the top external causes of TBIs in Chinese children. When compared with national data from the developed countries, gender patterns are similar, but the ranking of external causes is different. This is the first study to highlight the important role of suspected child abuse in causing TBIs in infants in China. TBIs caused by child abuse warrant further research and government attention as a social and medical problem in China.

## Introduction

Traumatic brain injury (TBI) is a leading cause of death and long-term disabilities around the world. [1] Previous studies indicate that TBIs can have a significant negative impact on the lives of the injured and their family members and inflict a huge economic burden on healthcare systems. [2], [3], [4], [5], [6], [7], [8], [9], [10] For these reasons, many countries have developed surveillance systems to measure the impact of TBIs and have conducted epidemiologic studies to identify risk factors and patterns of TBI to guide and evaluate effective prevention programs. [10], [11], [12], [13], [14], [15].

Results of previous studies suggest that males experience more TBIs than females and that children and the elderly have higher prevalence of TBIs than other age groups. [10], [16] The leading external causes of TBIs in the U.S. are, falls, road traffic collisions, struck by/against, and assault. [10] However, the leading external causes can differ across countries, and these differences are attributed to, among other factors, different socioeconomic circumstances. In high-income countries, TBIs caused by traffic collisions have been on the decline [17], while the incidence of TBIs caused by falls has been increasing because of the growing elderly population. [16]In low-income and middle income-countries, the incidence of TBIs is increasing rapidly due to significantly more road traffic collisions. [16], [18], [19] Child abuse is now being reported as an important cause of TBIs in children in high income countries. [20], [21].

A number of studies have been published to characterize traumatic brain injury in China. [22], [23], [24], [25] In 1983, a door-to-door epidemiological survey in six Chinese cities reported that traffic incidents, struck by/against objects, and falls were the main causes of head trauma. [23] Another large epidemiologic study (246,812 participants) conducted from 1983–1985 in 21 provinces of China compared causes of traumatic brain injuries between rural and urban areas. Traffic collisions, falls, stumbles and assaults were found to be the leading causes in urban and rural areas, but TBIs caused by assaults were more common in cities. [25] In a more recent study, 14,948 TBI cases treated at 77 hospitals in eastern China were studied, and similar leading causes of TBIs were reported. [24] Zhu et al. reported that farmers, labor workers, and children were more likely than other individuals to sustain TBIs. [25] All these previous studies from China reported that males have a higher incidence of TBIs than females.

Although some studies have investigated epidemiological characteristics and outcomes of TBIs in China, TBIs have not been investigated to the degree that an issue of this importance deserves, especially given the size of the population and the increase in the number of road traffic collisions. [18], [26] Additionally, TBIs in Chinese children have not received the same attention as adult TBIs. In 2002, Yang et al. reported on 1,300 pediatric TBI patients at a general hospital in China; approximately two thirds of the patients were from rural areas; falls and traffic collisions were the leading causes of pediatric TBI. [27] No publications were found that specifically address sports-related or abuse-related TBIs among children in China.

This study was undertaken to address gaps in the literature on the characteristics and causes of pediatric TBIs in China. We obtained the medical records for all TBI cases that resulted in hospitalization from a large metropolitan children’s hospital in China for the years 2002–2011. We sought to answer the following research questions: (1) are sports and suspected child abuse among the leading external causes of pediatric TBIs in China; (2) did the proportion of TBIs caused by road traffic collisions increase during the ten-year period of 2002–2011. We also studied the distribution of ages of pediatric TBIs caused by road traffic collisions, falls, and assault/child abuse. The underlying research hypothesis was that these three major causes of pediatric TBIs have distinct age patterns that could guide the development of TBI prevention programs among Chinese children.

## Methods

Our study was reviewed and approved by the Institutional Review Board of Tongji Medical College School of Public Health in Wuhan, China and the Research Institute at Nationwide Children’s Hospital in Columbus, Ohio.

### Data Source

Hospital discharge data from the Wuhan Medical Care Center for Women and Children (WMCCWC) were analyzed for this study. Wuhan is the capital city of Hubei Province, The People’s Republic of China. According to official statistics, Wuhan City had 9.8 million permanent residents, including 976,947 children aged 0–14 years, in 2010. [28] WMCCWC is the largest comprehensive women and children’s hospital in central China and it has been ranked as one of the best children’s hospitals in China. Supported by approximately 1360 medical staff and researchers, WMCCWC provides a full range of clinical services as well as health promotion and prevention for the women and children of Hubei and neighboring provinces. Two thirds of patients treated at WMCCWC live in cities and one third come from rural villages. WMCCWC currently has four outpatient departments and two inpatient buildings. WMCCWC has 1112 beds and treats approximately 30,000 inpatients and 900,000 outpatients at outpatient and emergency departments (ED) annually. According to the Wuhan Bureau of Health (personal communication), WMCCWC treats approximately 55–60% of total pediatric outpatients and ED patients in Wuhan City.

Data were extracted from the the medical records database of the WMCCWC. Medical records of all inpatients treated at the WMCCWC are reviewed and diagnosis codes are assigned by trained coders using the International Classification of Diseases 10^th^ Revision (ICD-10) [29]. The following variables were included in the database that was provided to the research team: medical record number, date of birth, gender, date of admission, admission diagnosis, discharge date, ICD-10 diagnosis code, type of condition (injury and poisoning or other), description of injury events and external causes, and outcomes at discharge.

### Study Population and TBI Case Definition

The study population was children 17 years or younger who were discharged as inpatients from the WMCCWC with a discharge diagnosis of traumatic brain injury during 2002–2011. Because medical records before 2002 were coded using the International Classification of Diseases, Ninth Revision, Clinical Modification (ICD-9-CM), earlier hospitalized cases were not considered for this analysis.

We utilized a list of ICD-10 codes used in U.S. studies of traumatic brain injuries [30], [31] to identify TBI cases in the medical records of the WMCCWC. The detailed codes are listed in Table 1. The outcomes at discharge are those reported in the medical record and are based on the assessment of the discharging physician. Simple outcome categories were available in the medical records database, but comprehensive functional assessments are not usually conducted for hospitalized TBI patients at the time of discharge.

**Table 1:** Diagnosis ICD-10 codes of inpatient traumatic brain injuries treated at a large urban children's hospital, Wuhan, China (2002–2011).

Description	ICD-10	Sample n	%
Total		4230	
Open wound of the head	S01.1–S01.9	1309	31.0
Fracture of the skull and facial bones	S02.0, S02.1, S02.3, S02.7–S02.9	489	11.6
Injury to optic nerve and pathways	S04.0	3	0.1
Intracranial injury	S06.0–S06.9	1669	39.5
Crushing injury of head	S07.0, S07.1, S07.8, S07.9	–	
Other unspecified injuries of head	S09.7–S09.9	759	17.9
Open wounds involving head with neck	T01.0	–	
Fractures involving head with neck	T02.0	–	
Crushing injuries involving head with neck	T04.0	–	
Injuries of brain and cranial nerves with injuries			
of nerves and spinal cord at neck level	T06.0	–	
Sequelae of injuries of head	T90.1, T90.2, T90.4, T.90.5, T90.8, T90.9	1	0.02

### External Cause of Injury

One of the researchers (Dr. Zhu at the Tongji Medical College School of Public Health) reviewed the medical record descriptions of the injury events for each TBI case. External causes were then assigned using the following major categories: traffic collisions (with subgroups of motor vehicle passengers, pedestrians, bicycle and tricycle riders, motorcycle riders, and other), falls, struck by/against objects, sports, assault and suspected child abuse, and other.

### Data Analysis

Data were analyzed using the SAS statistical software (SAS Institute Inc., Cary, NC). [32] We first provided the frequency and percentage distribution of the TBI cases by diagnosis groups. We provided descriptive statistics for gender, age, outcome, and length of hospital stay. External causes of TBIs were then compared by age groups: 0–2 years, 3–6 years, 7–12 years, and 13–17 years. This age classification was used based on developmental stages of children and the TBI risk associated with different developmental stages and activities. [33], [34] We graphed the frequency of TBI by age for the following causes: falls, traffic collisions, and suspected child abuse. We calculated the percentages (%) and 95% confidence intervals (95% CI) for traffic related TBIs for all inpatient pediatric TBIs for each year.

## Results

A total of 4,230 pediatric TBI patients were hospitalized at the WMCCWC during 2002–2011. Table 1 provides the frequency and percentage distributions of TBIs by ICD-10 diagnosis groups. Intracranial injury (39.46%), open wound of head (30.95%), crushing injury of head (17.94%), and fracture of the skull and facial bones (11.56%) accounted for 99.9% of pediatric TBIs. Less than 10 TBI cases were seen in each of the following two categories: ‘Injuries to optic nerves and pathways’ and ‘Sequelae of injuries to head’.

Table 2 describes the characteristics of patients and their outcomes. The percentage of cases of TBIs among males (65.2%, 95% CI: 63.7%–66.6%) was higher than among females (34.8%, 95% CI: 33.4%–36.3%) with a ratio of 1.87∶1. About half of TBI patients were children 0–2 years old (50.1%: 95% CI: 48.5%–51.6%), and 34.5% (95% CI: 33.1%–36.0%) of patients were children 3–6 years old. More than two thirds (67.1%, 95% CI: 65.4%–68.8%) of patients were residents of urban cities, and 28.8% (95% CI: 26.3%–31.3%) came from rural villages. Approximately three-fourths (72.2%) of TBI patients experienced a full recovery, and one-fourth (25%) had a partial recovery at time of hospital discharge, based on the assessment of the discharging physician. The mean hospital stay was 7.2 days with a minimum of less than one day and a maximum of 167 days.

**Table 2:** Characteristics of inpatient pediatric traumatic brain injuries treated at a large urban children’s hospital, China, 2002–2011.

Gender*			
Male	2756	65.2	(63.7–66.6)
Female	1473	34.8	(33.4–36.3)
Age (years)			
0–2	2117	50.1	(48.5–51.6)
3–6	1460	34.5	(33.1–36.0)
7–12	599	14.2	(13.1–15.2)
13–15	47	1.1	(0.8–1.5)
16–18	7	0.2	(0.1–0.3)
Urban/rural location of residence			
Urban	2837	67.1	(65.4–68.8)
Rural	1222	28.8	(26.3–31.3)
Unknown	171	4.1	(1.7–7.6)
Outcomes			
Full recovery	3055	72.2	(70.9–73.6)
Partial recovery	1058	25	(23.7–26.3)
Death	14	0.3	(0.2–0.5)
Unknown	103	2.4	(2.0–2.9)
Length of stay (days)*			
Mean (S.D.)		7.2 (6.0)	
Min-Max		0.2–167	

* Missing information for one record.

S.D. = Standard deviation.

Table 3 provides the external cause categories of the TBIs cases. For all age groups, falls, struck by/against objects, and traffic collisions were the leading external causes of TBIs. Falls caused about half of TBIs in children 0–2 years and 3–6 years. The proportion of TBIs caused by traffic collisions ranged from 10.1% in children 0–2 years old to 18.6% in 3–6 year olds. Among the TBI cases caused by traffic collisions, the largest proportions were among motor vehicle passengers, bicycle/tricycle riders, and motorcycle riders, and each of these categories accounted for about 20% of the traffic crash related TBIs in each age group. There were 125 TBIs in children 0–2 years of age (5.9% of all TBIs were in this age group) that were caused by suspected child abuse.

**Table 3:** External cause of inpatient pediatric traumatic brain injuries treated at a large urban children’s hospital,China, 2002–2011.

External Cause	0–2 Years		3–6 Years		7–12 Years		13–17 Years		All ages	
	n	%	n	%	n	%	n	%	n	%
Total	2117		1460		599		54		4230	
Traffic collisions	214	10.1	271	18.6	100	16.7	8	14.8	593	14.0%
Passengers	49	22.9	54	19.9	21	21.0	2	25.0	126	21.2%
Pedestrians	8	3.7	9	3.3	3	3.0	0	–	20	3.4%
Bicycles/tricycles	41	19.2	56	20.7	26	26.0	4	50.0	127	21.4%
Motorcycles	47	22.0	60	22.1	22	22.0	0	–	129	21.8%
Other	69	32.2	92	33.9	28	28.0	2	25.0	191	32.2%
Falls	1231	58.1	702	48.1	268	44.7	17	31.5	2218	52.4%
Struck by/against objects	459	21.7	411	28.2	187	31.2	15	27.8	1072	25.3%
Sports	5	0.2	15	1.0	9	1.5	1	1.9	30	0.7%
Assault and child abuse	125	5.9	15	1.0	19	3.2	8	14.8	167	3.9%
Other causes	83	3.9	46	3.2	16	2.7	5	9.3	150	3.5%

**Figure 1:**
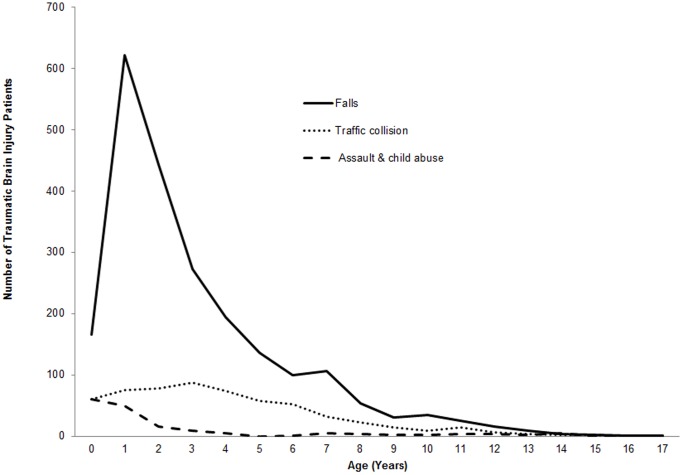
Age patterns of three major causes of pediatric traumatic brain injuries treated at a large urban children’s hospital in China, 2002–2011.

Figure 1 illustrates the distribution by age of TBIs caused by falls, traffic collisions, and assault/suspected child abuse. These results show the unique patterns by age for the major external causes of pediatric TBIs. Falls peaked at age 2 and were responsible for the greatest number of TBIs especially among younger children. TBIs as a result of traffic collisions peaked at age 3. Suspected child abuse related TBI cases were more common among children 0–1 year of age.

**Figure 2:**
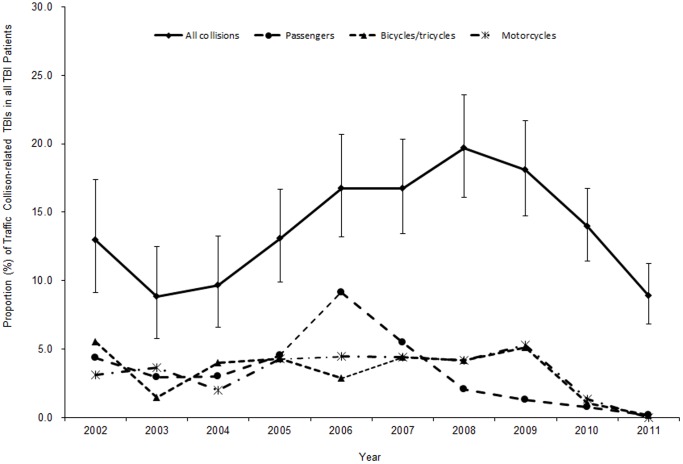
Trend of propotion of traffic-related brain injury patients in all pediatric traumatic brain injury patients treated at a large urban children’s hospital in China, 2002–2011.

Figure 2 shows the proportion of pediatric TBIs caused by traffic collisions by year. The proportion of TBIs caused by traffic collisions among hospitalized pediatric TBI patients increased from 12.99% (95% CI: 9.15%–17.40%) in 2002 to 19.68% (95% CI: 16.07%–23.55%) in 2008. After 2008, the proportion of TBIs caused by traffic collisions began decreasing, reaching 8.91% (95% CI: 6.82%–11.23%) in 2011.

## Discussion

Previous Chinese epidemiological studies have examined demographic patterns and risk factors of TBI patients. [23], [24], [25] The findings of our study are consistent with previous research which found that males represent a higher percentage of hospitalized TBI patients than females. Zhao et al. reported that the male-female ratio was 1.7 to 1 in cities and 2.5 to 1 in rural areas. [25] Wu et al. found that of the 14,948 TBI patients treated at 77 hospitals in eastern China, 76.6% were males and 25.4% were females. [24] In a population-based survey, Wang et al. reported that the age-adjusted prevalence per 100,000 residents was 883 for males and 536 for females. [23] International studies of pediatric TBIs also reported that males experience TBIs at a disproportionate rate to females (60% vs. 40% of total TBIs cases). [35] In the U.S., major risk factors for TBIs are gender, age, and socioeconomic status. [10], [31], [36] The male predominance in TBI cases could be attributed to greater risk-taking behavior and high-risk activities among males. [10].

All three previously described Chinese studies reported that road traffic injury is the leading external cause of TBIs in China. [23], [24], [25] Although these three previous studies included all age groups, they do not provide separate analyses of external causes for children and adolescents. In contrast to these three previous Chinese studies, we found falls were the leading cause of TBI among children. In a study among hospitalized pediatric TBI patients at a general hospital in China, falls were also the leading cause, accounting for 50.46% of TBIs. [27] When compared with pediatric national data from the U.S., the ranking of external causes is different. [37] In the U.S. in 2006, motor vehicle collisions caused about 40% of pediatric hospitalizations, and falls caused about 20%; assault was the third leading cause. [37]. Additionally, in the U.S., rates of TBI hospitalization caused by motor vehicle collisions increased significantly after age 15 and peaked at age 19. [37] In China, one must be 18 years or older to get a driver’s license. This fact possibly explains why so few traffic related TBIs are reported among those 13–17 years of age in our study. Additionally, family car ownership is a relatively new phenomenon in China; thus, getting a driver’s license and driving the family car by teenagers are not yet very common. Another possible reason for the different patterns of TBIs caused by traffic collisions between our study and U.S. studies is that older kids are more likely to receive medical treatment at other local hospitals than at a children’s hospital. A national study of pediatric head trauma patients who are treated at different types of hospitals may provide better evidence.

With rapid economic development and improved standards of living, the percentage of Chinese households with cars has increased dramatically in the past decade. Consequently, during the same period, traffic related injuries have increased significantly. [18] Our previous study based on inpatient medical records at this medical center reported that the proportion of hospitalizations for injuries caused by motor vehicle-pedestrian collisions almost doubled from 1.6% in 1993 to 3.1% in 2004. [38] Results reported in this current study indicate that the proportion of traffic related TBIs out of all pediatric TBIs increased from 2002 to 2008 but declined each year after 2008. A similar reduction in the number of traffic related deaths after 2008 in China was seen in a study utilizing official police reporting. [18] However, some researchers suggest that traffic related deaths based on police documentation are severely underreported in China. [39] Fortunately, our data came from the medical records of a large medical center, and we do not believe that physicians have any reason to underreport traffic related TBIs. We assert that traffic related pediatric TBIs warrants continued, unbiased surveillance using data other than police collision reports. .

Our study is consistent with findings in the U.S. in relation to TBIs caused by suspected child abuse in young children. [20], [33], [34] TBIs caused by suspected child abuse were significantly more likely to occur in children 0–1 year of age than in older age groups. In the 10 years included in our study, suspected child abuse caused 125 TBIs in infants. Previous studies in the U.S. suggested that pediatric TBIs caused by child abuse were associated with more severe diagnoses and negative outcomes. [33], [40], [41] Although previous studies have addressed assault-related head trauma in China [42], [43], [44] and one study concluded that assault-related head trauma was “*increasing and becoming both a social and medical problem in China*,” [42] we did not find any publications addressing child abuse as a major cause of TBIs in Chinese infants. The assault related head traumas examined in previous publications were injuries caused by interpersonal violent events that are more frequently reported for young adults. [42] Unlike the child abuse reporting requirements in the U.S., to our knowledge, there is no policy requiring a physician to report suspected child abuse to Chinese authorities. In our study, if the physician suspected that the TBI was caused by child abuse, he or she wrote a note in the medical records. No further legal reporting is required in medical practice in China. Due to this lack of required reporting, it is very likely that TBIs caused by child abuse were under-reported in our study. Furthermore, we could only report these cases as “suspected child abuse” because a systematic legal procedure for confirming suspected child abuse cases does not exist in China. Reporting child abuse in the medical care of pediatric TBIs in China may prove to be a complex issue given deep-rooted sociocultural factors and the current legal system in China. Nevertheless, child abuse as a major cause of TBIs in infants must be studied further to raise awareness of this important social and medical problem in China.

This study is the first from China to use TBI definitions based on ICD-10 diagnostic codes. [23], [24], [25] Using ICD-10 codes to identify TBI cases allows a comparison of our findings with results of previous international research. [30], [31], [34] The Wuhan Medical Care Center for Women and Children is charged with two major roles: a) clinical care of women and children; and b) preventive programs targeting children in Wuhan City. This dual-role model in a metropolitan medical center provides a good opportunity to translate research findings from clinical care into prevention programs in the local community.

Interpretation of our results should take into consideration several limitations of our study. First, the data should not be considered a probability sample of pediatric TBIs in Wuhan, nor were we able to calculate injury rates. We only evaluated hospital discharge data from one large children’s medical center. Second, the hospital discharge data at the WMCCWC lack many important data elements for TBI outcome assessments. For example, the available simple outcome variable contains only the subjective assessment provided by the discharging physician. It is possible that this could have introduced bias. The Interagency Traumatic Brain Injury (TBI) Outcomes Workgroup has recommended outcome measures, but these have not been widely adopted in China. [45], [46] Unlike the medical records data that are used for insurance claim purposes by hospitals in the U.S., there are not requirements for minimum data elements to be collected by hospitals. Electronic hospital medical records in China do not contain comprehensive treatment and outcome information. Future TBI studies in China need to use standard outcome measures to assess TBI outcomes [45], [46] and disabilities caused by TBI in Chinese children. [47] TBI researchers and national medical associations need to work together to develop standard TBI outcome assessment tools and hospitals must have incentives and a good reason to collect these data.

In conclusion, this study found that falls, struck by/against objects, and traffic collisions are the leading external causes of pediatric TBIs, but this study also highlights the important role of suspected child abuse in causing TBIs in infants in China. Falls, traffic collisions, and suspected child abuse have unique associated age patterns so that different prevention programs should be developed to target different age groups. Our finding of suspected child abuse as an important external cause of TBIs in infants is important because previous publications from China have not considered this issue. TBIs caused by child abuse warrant further research and government attention as a social and medical problem in China.
